# Cannabinoids—Multifunctional Compounds, Applications and Challenges—Mini Review

**DOI:** 10.3390/molecules29204923

**Published:** 2024-10-17

**Authors:** Dominik Duczmal, Aleksandra Bazan-Wozniak, Krystyna Niedzielska, Robert Pietrzak

**Affiliations:** 1 Department of Applied Chemistry, Faculty of Chemistry, Adam Mickiewicz University in Poznań, Uniwersytetu Poznańskiego 8, 61-614 Poznań, Poland; dominik.duczmal@amu.edu.pl; 2 Polygen Sp. z o.o., Górnych Wałów 46/1, 44-100 Gliwice, Poland; krystynan@polygen.com.pl

**Keywords:** cannabinoids, cannabis, THC, CBD, cannabis-based medicines, biological activities

## Abstract

Cannabinoids represent a highly researched group of plant-derived ingredients. The substantial investment of funds from state and commercial sources has facilitated a significant increase in knowledge about these ingredients. Cannabinoids can be classified into three principal categories: plant-derived phytocannabinoids, synthetic cannabinoids and endogenous cannabinoids, along with the enzymes responsible for their synthesis and degradation. All of these compounds interact biologically with type 1 (CB1) and/or type 2 (CB2) cannabinoid receptors. A substantial body of evidence from in vitro and in vivo studies has demonstrated that cannabinoids and inhibitors of endocannabinoid degradation possess anti-inflammatory, antioxidant, antitumour and antifibrotic properties with beneficial effects. This review, which spans the period from 1940 to 2024, offers an overview of the potential therapeutic applications of natural and synthetic cannabinoids. The development of these substances is essential for the global market of do-it-yourself drugs to fully exploit the promising therapeutic properties of cannabinoids.

## 1. Introduction

**Figure 1:**
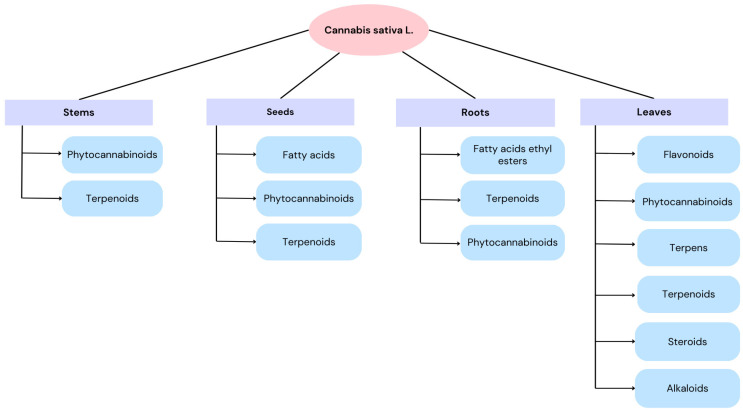
Chemical composition of various parts of *Cannabis sativa* L.

*Cannabis sativa* L. is an herbaceous plant in the family Cannabaceae, commonly known as marijuana or hemp. The plant is distinctive in its chemical composition and physiological properties, producing in excess of 560 different compounds. These include terpenes, alkaloids, phenols, flavonoids, amino acids, stilbenoids, fatty acids, carbohydrates and hydrocarbons (Figure 1). Despite its origins in Central Asia, the species has demonstrated remarkable adaptability, allowing it to flourish in diverse climatic conditions across the globe. The Cannabis genus comprises a single species, *Cannabis sativa*, which is further divided into several subspecies or varieties. The precise classification of these subspecies and varieties remains a topic of debate within the scientific community. *Cannabis sativa* L. is one of the oldest cultivated plants, with evidence of human use dating back thousands of years. It has been employed as a source of fiber, food and oil and used for religious purposes [1,2,3,4,5]. The cannabinoids discovered in *Cannabis sativa*, such as Δ-9-tetrahydrocannabinol (THC), cannabidiol (CBD), cannabinol (CBN), cannabigerol (CBG) and cannabichromen (CBC), represent a diverse group of bioactive compounds that interact with the human endocannabinoid system, each exhibiting distinct pharmacological effects, ranging from psychoactivity and pain relief to anti-inflammatory and neuroprotective properties, making them subjects of increasing scientific and medical interest.

**Figure 2:**
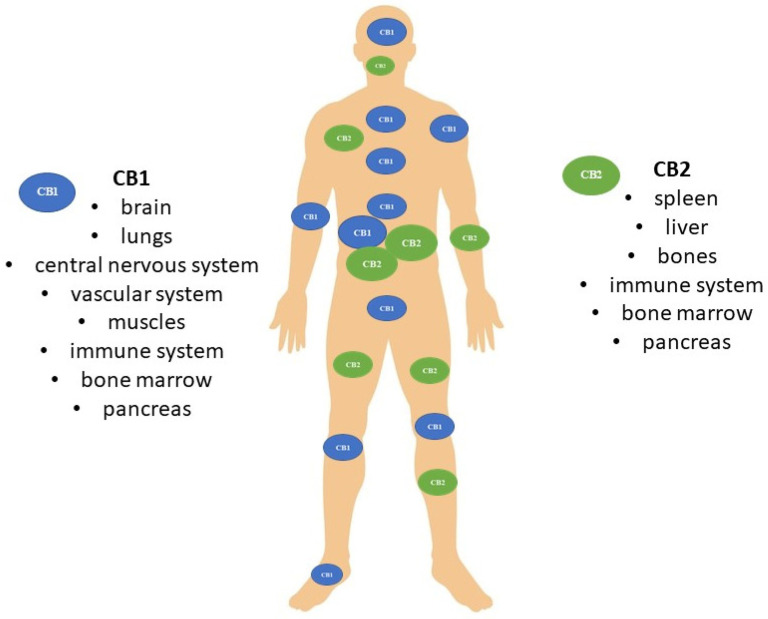
Distribution of cannabinoid receptors CB1 and CB2 in the human body.

The discovery of the CB1R and CB2R (Figure 2) receptors in the human body in the 1990s facilitated a more comprehensive understanding of the relationship between cannabinoids and the human body, particularly CB1R, which is the primary receptor subtype of the central nervous system. Subsequent pharmacological studies have demonstrated the considerable potential of this compound in a variety of therapeutic applications. It has been employed in the treatment of numerous conditions and ailments, including the management of epilepsy seizures, cancer-related pain, anxiety, depression, mood disorders and addictions to alcohol, nicotine and post-traumatic stress disorder [1].

Cannabinoids exert effects similar to certain alkaloids, such as morphine and nicotine, significantly influencing the neurons of the central nervous system by modulating pain, mood and cognitive functions. However, in contrast to alkaloids, cannabinoids lack a heterocyclic nitrogen atom, which precludes their classification within this category of biologically active compounds. Nevertheless, they do feature an oxygen atom within their heterocyclic system, exhibiting analogous physicochemical properties to those of the nitrogen atom. Consequently, their biological impact exhibits partial similarities to that observed in alkaloids such as caffeine, morphine, nicotine and cocaine [6,7,8,9,10].

The classification of cannabinoids is based on their chemical structure and action in the body. The most well-known group are the plant cannabinoids, which are produced by the cannabis plant. These compounds, known as phytocannabinoids, include substances such as Δ-9-tetrahydrocannabinol (THC), cannabidiol (CBD), cannabinol (CBN) and many others. Each of these cannabinoids has unique properties and potential therapeutic uses. The next group of cannabinoids are those produced internally by the human body, known as endocannabinoids [11,12,13,14,15]. The most important of these are anandamide (AEA) and 2-arachidonoylglycerol (2-AG) [16,17,18,19,20,21,22,23,24]. These compounds play a key role in regulating many physiological processes, interacting with cannabinoid receptors in the body to affect various aspects of health and well-being. Synthetic cannabinoids are chemical compounds created in laboratories that do not occur naturally in the cannabis plant. These compounds include both AC-bicyclic and ACD-tricyclic cannabinoids, such as CP55940. CP55940 is a synthetic analogue of Δ9-THC (delta-9-tetrahydrocannabinol) and has played a pivotal role in identifying cannabinoid receptors. The use of such substances has facilitated further exploration and understanding of the mechanisms of action of the cannabinoid system [25,26,27].

**Figure 3:**
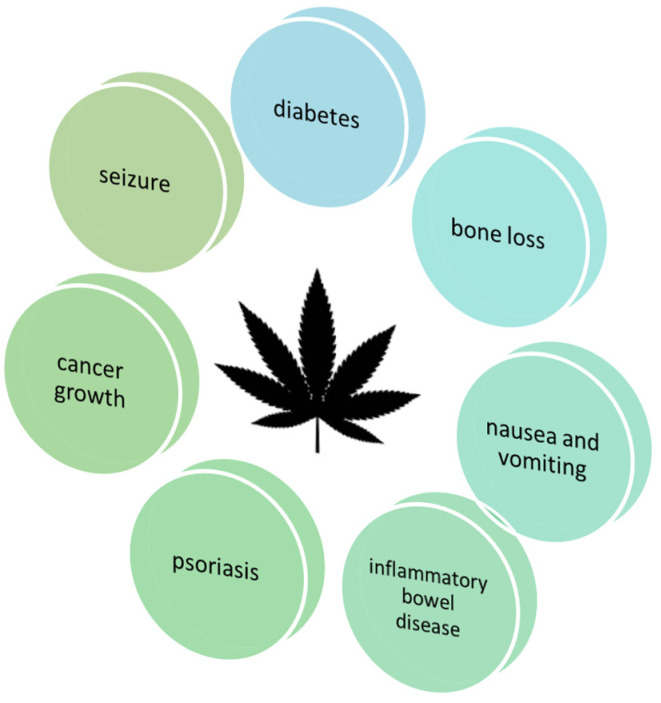
Medical use of cannabis.

The global pharmaceutical industry is increasingly recognizing the therapeutic potential of cannabis and the health benefits of its active constituents, known as cannabinoids. These compounds have demonstrated efficacy in the management of chronic pain, the reduction in muscle spasticity in individuals with multiple sclerosis and the treatment of nausea and vomiting associated with chemotherapy. Additionally, cannabinoids are employed in the management of refractory epilepsy, as well as in the treatment of depression, anxiety, sleep disorders and rheumatic diseases [28,29,30,31,32,33,34,35]. In this review, we have gathered information on the most popular cannabinoids, outlining their biological properties, potential uses and the challenges faced by the market offering these compounds (Figure 3).

## 2. Method

In order to identify pertinent articles for this review, a combination of search terms, including “cannabinoids”, “cannabis”, “medicinal cannabis” and “analytics”, was employed across four databases: PubMed, ScienceDirect, Google Scholar and the Cochrane Library (Wiley).

## 3. Primary Cannabinoids and Their Effects

### 3.1. Phytocannabinoids

#### 3.1.1. ∆-9-Tetrahydrocannabinol (THC)

**Figure 4:**
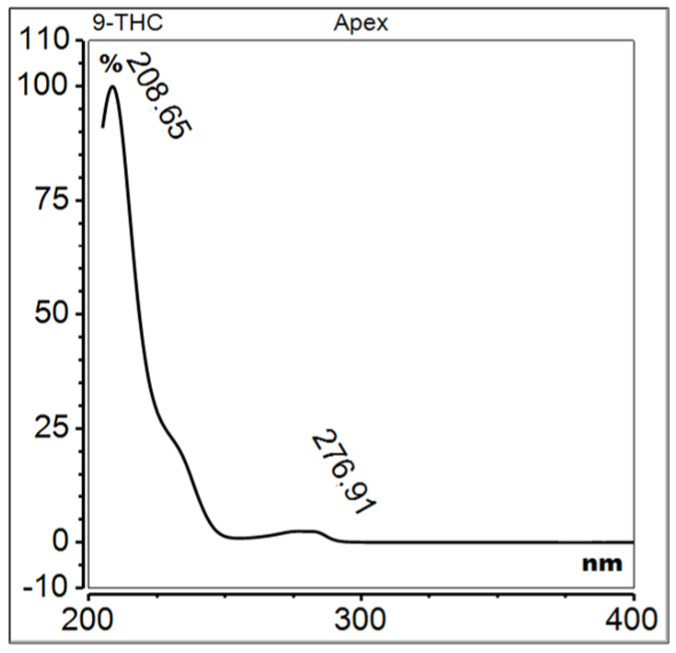
UV spectrum of Δ9-tetrahydrocannabinol.

**Figure 5:**
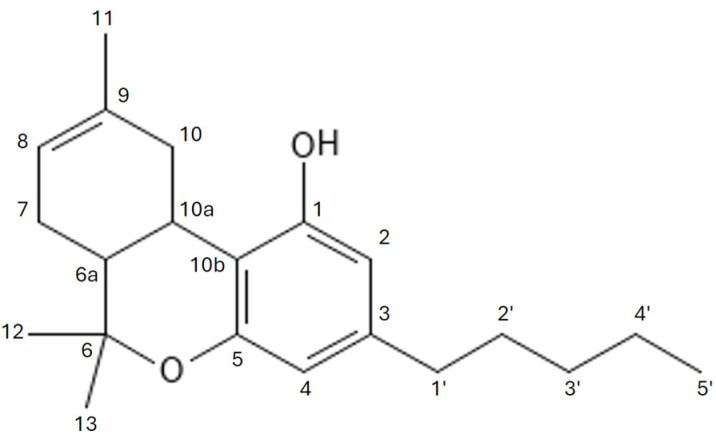
Chemical structure of ∆-8-tetrahydrocannabinol.

**Figure 6:**
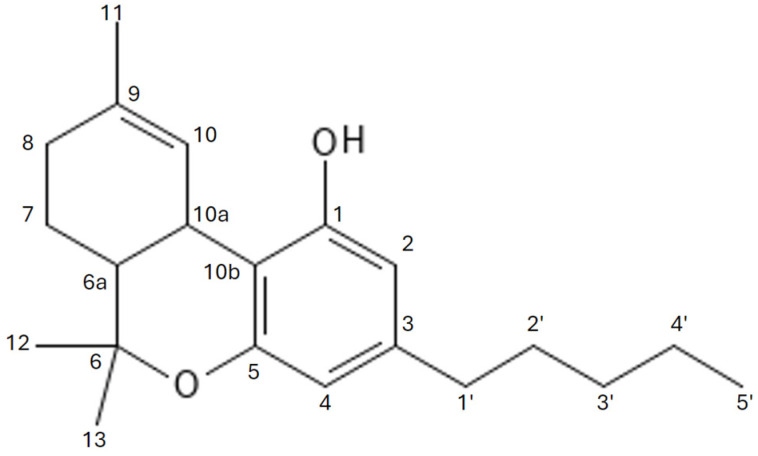
Chemical structure of ∆-9-tetrahydrocannabinol.

The primary phytocannabinoid present in cannabis is Δ9-THC IUPAC name 6,6,9-Trimethyl-3-pentyl-6a,7,8,10a-tetrahydrobenzo[c]chromen-1-ol, whose chemical structure was initially synthesized and described by Rafael Mechoulam in 1964 [36] (Figure 4). This molecule is formed by the decarboxylation of tetrahydrocannabinolic acid (THCA), which itself lacks psychoactive properties. THCA is the end-product of a biosynthetic pathway in which cannabigerolic acid (CBGA) is converted by the enzyme THCA synthase. It is notable that Δ9-THC is less stable than its isomer Δ8-THC and is prone to undergoing transformations such as isomerization of the double bond or elimination of a hydrogen atom from position 10a (Figure 5 and Figure 6). These transformations result in the formation of conjugated dienes, which are likely to be a precursor to aromatic CBN. In its natural form, Δ9-THC exists as a mixture of two precursor carboxyl forms, Δ9-THCA and Δ9-THCB [37]. Of particular note is the potent neuroprotective effect of Δ9-THCA in its carboxyl form, which may prove invaluable in the context of neurodegenerative and neuroinflammatory disease treatment [38].

Tetrahydrocannabinol (THC) is acknowledged as the principal psychoactive constituent of cannabis. It functions as a partial agonist of the cannabinoid type 1 (CB1) and type 2 (CB2) receptors within the endocannabinoid system [39]. THC functions as an agonist for several receptors, including PPARα-γ, GPR55, GPR18, TRPV2-4 and TRPA1. Additionally, it acts as an antagonist for subfamily M TRP cation channels (TRPM8) and serotonin 3A (5-HT3A) receptors [40,41]. Synthetic THC, marketed under the brand name Marinol^®^, has been approved by the United States Food and Drug Administration for the treatment of nausea and vomiting associated with cancer chemotherapy [42]. In recent years, the use of synthetic THC-based prescription drugs, such as nabiximols, dronabinol and nabilone, has increased for the treatment of neurological disorders [43]. Δ9-Tetrahydrocannabinol possesses potential therapeutic benefits, including pain relief, nausea and vomiting control, appetite stimulation and anti-inflammatory effects [44].

It should be noted that the adverse effects associated with cannabis are primarily attributable to THC. Therefore, the total daily equivalent dose of THC should generally be limited to 30 mg/day or less, preferably in combination with CBD. In contrast to medical cannabis, recreational cannabis typically contains high concentrations of THC to achieve a euphoric effect [45]. The abuse of cannabis during adolescence, particularly when high doses are consumed over an extended period, has been demonstrated to elevate the likelihood of developing psychotic symptoms in later life [46]. The findings of the study, as detailed in the paper [47], indicate that there is an elevated risk of suicidal behavior and the emergence of manic symptoms among patients with bipolar disorder who engage in heavy cannabis use.

#### 3.1.2. Cannabidiol (CBD)

**Figure 7:**
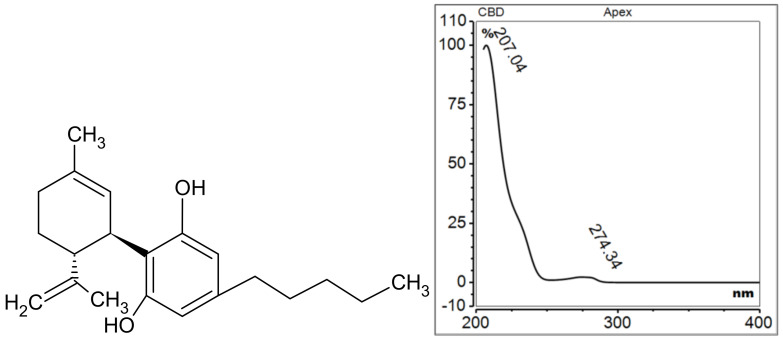
Chemical structure and UV spectrum of cannabidiol.

Cannabidiol (CBD) IUPAC name 2-[(1R,6R)-3-methyl-6-prop-1-en-2-ylcyclohex-2-en-1-yl]-5-pentylbenzene-1,3-diol (Figure 7) is one of the primary phytocannabinoids found in *Cannabis sativa*. Unlike Δ9-tetrahydrocannabinol (∆9-THC), another compound from cannabis, CBD does not produce intoxication or display the typical characteristics associated with substances of abuse. CBD is a phenolic monoterpene that was initially extracted by Adams, Hunt and Clark in 1940 from Mexican marijuana (*Cannabis sativa* L.) and subsequently by Jacob and Todd from the resin of *Cannabis indica* [48,49,50]. CBD has been demonstrated to exert a range of comprehensive biological effects, including antioxidant and anti-inflammatory properties [51]. A review of the literature indicates that cannabinol is being considered as a potential treatment for a number of conditions, including diabetes, cardiovascular disease, cancer, arthritis, psychosis, epilepsy, Alzheimer’s disease and various skin conditions [51,52,53].

The bioavailability of CBD is largely contingent upon the method and route of administration. In the majority of cases, CBD is available in solution (either oil or alcohol), which is then converted into gelatin capsules, oral solution or sublingual drops [54,55].

Cannabinoids exert a dual effect on the immune system, with both positive and negative consequences. CBD has been demonstrated to reduce inflammation, which is a natural response of the immune system to injury. However, chronic inflammation can contribute to the development of numerous diseases [56]. CBD exerts its anti-inflammatory effects through mechanisms that are related to both innate and adaptive immune responses. It exerts its effects by inhibiting the production of inflammatory cytokines, reducing the secretion of nitric oxide and pro-myeloperoxidase by innate cells. Furthermore, CBD inhibits inducible nitric oxide synthase, mediates lymphocyte apoptosis, which contributes to the immunosuppressive effect, and reduces the activation of microglia cells. Additionally, CBD influences the activity of antioxidant and pro-oxidant enzymes, promotes the chelation of transition metal ions and modifies oxidative processes involving lipids, proteins and DNA [56,57]. Additionally, CBD has been the subject of investigation in the context of autoimmune diseases. There is evidence that CBD may modulate the immune response in such conditions, potentially reducing symptoms and improving quality of life. However, research on CBD and the immune system is still in its infancy and requires further study [57].

Animal and cell culture studies have demonstrated that CBD has the potential to lower blood pressure and improve blood flow. Additionally, CBD has been shown to reduce the risk of heart attack and stroke. Some research suggests that CBD might also lower the risk of atherosclerosis and offer beneficial effects on the cardiovascular system, especially in the context of obesity-related heart disease. While further studies are needed to fully understand CBD’s mechanisms of action on the cardiovascular system, it is important to note that, despite its promising potential, CBD should not be considered a substitute for conventional medical treatments [58,59].

CBD has been demonstrated to be safe; however, its direct use is questionable due to its poor solubility and low permeability. Nevertheless, these constraints can be surmounted by employing lipid and polymeric carriers, which enhance the solubility and permeability of CBD across diverse biological barriers, thereby facilitating improved bioavailability. In clinical trials assessing the efficacy of CBD in the treatment of various conditions, single doses are frequently employed, though the quantity may fluctuate contingent on the disease in question. Before recommending CBD to patients, further clinical trials are required to investigate the impact of regular multiple doses over an extended period. Nevertheless, patients frequently seek unproven over-the-counter CBD products for the treatment of various psychiatric and neurological conditions. Such practices should be monitored at a grassroots level, and public awareness of CBD use must be raised accordingly [57,60,61].

#### 3.1.3. Cannabinol (CBN)

**Figure 8:**
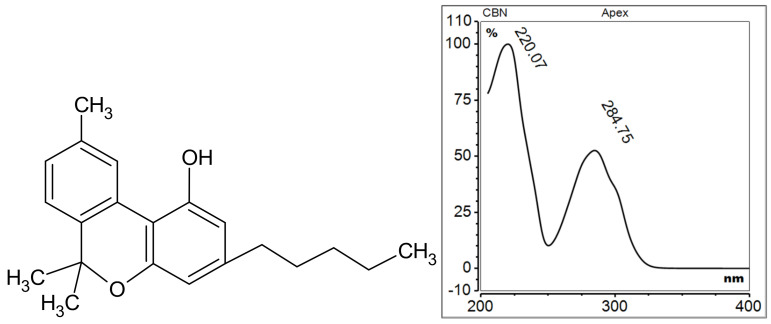
Chemical structure and UV spectrum of cannabinol.

Cannabinol, IUPAC name 6,6,9-trimethyl-3-pentylbenzo[c]chromen-1-ol, was first identified in the Cannabis sativa L. plant in 1930. It is a degradation product of Δ9-THC, resulting from an oxidation reaction that leads to the displacement of the double bond in the Δ9-THC molecule, which ultimately forms a fully aromatic structure [62] (Figure 8). Given its close structural relationship to CBD, CBN displays analogous properties, including anticonvulsant and anti-inflammatory effects [63].

The primary action of CBN is sedation and relaxation, making it a promising natural sleep aid. Its analgesic properties indicate a potential use in the treatment of pain, especially neuropathic and inflammatory pain [63]. CBN has only about 10% of the psychoactivity of THC, which makes it an interesting compound from a medical perspective. In vivo studies have indicated that cannabis may interact with signaling pathways that are involved in the growth and spread of cancer cells [64].

#### 3.1.4. Cannabigerol (CBG)

**Figure 9:**
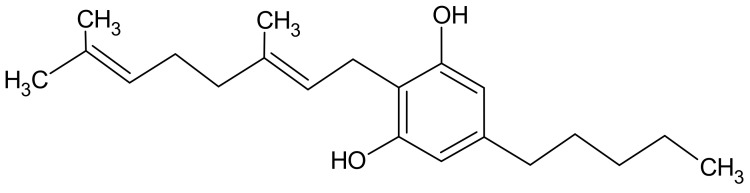
Chemical structure of cannabigerol.

**Figure 10:**
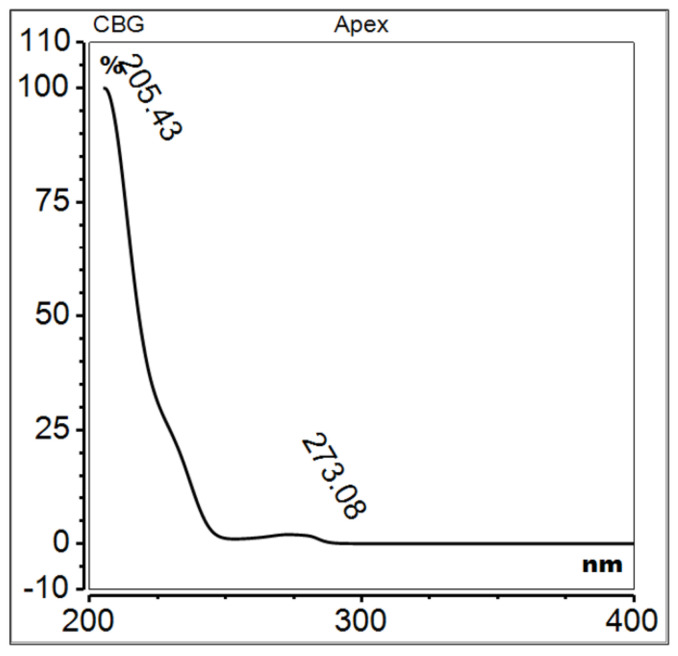
UV spectrum of cannabigerol.

Cannabigerol, IUPAC name 2-[(2E)-3,7-dimethylocta-2,6-dienyl]-5-pentylbenzene-1,3-diol (Figure 9 and Figure 10), has recently garnered significant interest from the field of pharmacology, primarily due to its non-psychotropic nature and its high content in certain strains of industrial hemp. In contrast to the more commonly encountered tetrahydrocannabinol (THC), CBG does not induce intoxication. Its potential benefits for human health include anti-inflammatory, analgesic, anticancer and neuroprotective effects [65].

The initial isolation of this compound occurred in 1964, utilizing a hexane extract of hashish. Subsequently, its structure and stereochemistry were confirmed through chemical synthesis. CBG plays a role as a precursor for other phytocannabinoids, including THC, CBD and CBC, in the process of biosynthesis. Given its unique properties and potential health benefits, CBG is the subject of intense research to understand its mechanisms of action and therapeutic applications [66,67].

It can be argued that commercially available CBG represents the fundamental basis for all cannabinoids, given its role as a precursor in the cannabis plant. It is important to note, however, that once absorbed by the human body, CBG does not undergo conversion into other cannabinoids. It is only the cannabis plant that is able to convert CBG into other cannabinoid molecules; this is not a capability that is present in human organisms. Furthermore, only a small number of cannabis varieties actually contain high concentrations of CBG. As a result, there are few reports in the scientific literature on the side effects of CBG in humans. Due to the limited information available, CBG remains the subject of research into its potential therapeutic use and possible side effects [67].

Cannabigerol (CBG) has demonstrated efficacy in the inhibition of unwanted biofilm formation and the eradication of pre-formed biofilms in antibiotic-resistant bacteria, including methicillin-resistant *Staphylococcus aureus* (*MRSA*) [68]. Researchers, including Jentsch and colleagues, have successfully synthesized CBG in a single step from inexpensive precursors such as 5-alkylresorcinols, olivetol and orcinol. In doing so, they have retained regioselectivity through the innovative use of alumina-promoted regioselective aromatic allylation reactions. However, the mechanism of this process is not yet fully understood, which is driving further research into reactive selectivity in cannabinoid synthesis [69]. The potential therapeutic applications of CBG and its derivatives (both natural and synthetic) have been the subject of recent research. These studies have explored the possibility of using these compounds to alleviate the adverse effects of chemotherapy and treat mood disorders (including depression), neurodegenerative diseases and nervous system diseases. Furthermore, their anesthetic effects have been the subject of extensive study. These studies indicate the multifaceted therapeutic potential of CBG, which may offer benefits in various areas of medicine, including the improvement of quality of life in patients undergoing chemotherapy and the treatment of serious neurological and psychiatric disorders [70,71].

CBG appears to exert an antagonistic effect on the antiemetic properties of THC, specifically through its interaction with CB1R and 5-HT1A receptors. This interaction has been observed to abolish the antiemetic effect of low doses of CBD, which is likely to be due to an effect on the 5-HT1A receptor. The potential of CBG to antagonize the antiemetic effects of the 5-HT1A receptor antagonist, 8-OH-DPAT, was also investigated. The objective of the experiments conducted on *Suncus murinus* (Japanese shrews) was to evaluate the potential effects of CBG in regulating nausea and vomiting. The results of these studies demonstrated that CBG antagonized the antiemetic effects of 8-OH-DPAT. Injection of moderate doses of CBG and CBD may produce opposing effects at the 5-HT1A receptor level, which interferes with the modulation of nausea and vomiting. These findings suggest that CBG may be a potential therapeutic agent due to its potential interaction with CBD, resulting from its opposing effects on the 5-HT1A receptor in the context of nausea and vomiting [72,73,74,75,76].

Further research is required on CBG, as it has the potential to be used, both alone and in combination with other substances, in new therapeutic approaches for various disorders. The evidence of CBG’s effects is currently derived primarily from preclinical studies, which indicate a promising outlook but also highlight the necessity for further clinical trials. The confirmation of the efficacy and safety of CBG in humans could facilitate the exploration of novel avenues for the treatment of conditions such as mood disorders, neurodegenerative diseases and the adverse effects of chemotherapy. It is imperative to pursue further research to fully elucidate the therapeutic potential of CBG and to ascertain the optimal utilization of its properties in clinical practice.

### 3.2. Endocannabinoids

#### 3.2.1. Anandamide (AEA)

**Figure 11:**
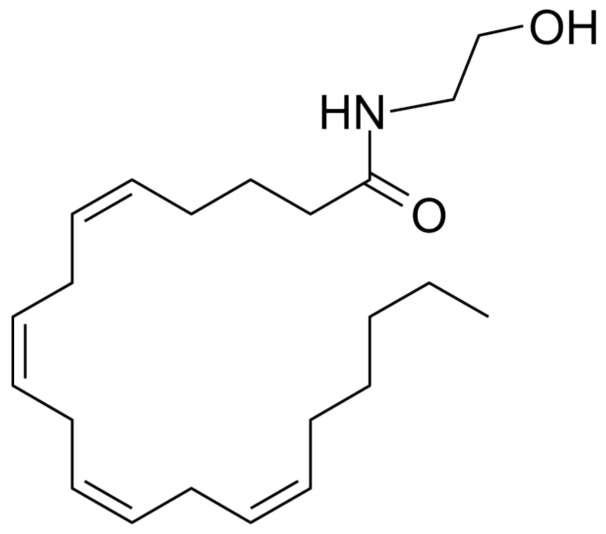
Chemical structure of anandamide.

Anandamide, IUPAC name (5Z,8Z,11Z,14Z)-N-(2-hydroxyethyl)icosa-5,8,11,14-tetraenamide (Figure 11), an endocannabinoid (eCB), is a neurotransmitter derived from fatty acids. It exhibits high permeability across the blood–brain barrier and exerts its effects on two principal cannabinoid receptor types: CB1 and CB2 [77,78]. Anandamide (AEA), first identified in 1992, remains one of the most well-known endogenous cannabinoids. It belongs to the large family of N-acyl ethanolamines (NAEs) and acts as a ligand agonist at both types of endocannabinoid receptors, with greater affinity for the CB1 receptor than CB2. AEA is produced in the body in small amounts through various biosynthetic pathways, involving the relevant enzymes [79,80,81].

The biosynthesis and metabolism of anandamide are carefully regulated by specific enzymes. The key enzyme involved in its biosynthesis is NAPE-PLD (N-acylphosphatidylethanolamine-specific phospholipase D), which catalyzes the hydrolysis of N-arachidonoylphosphatidylethanolamine (NAPE) to produce anandamide and phosphatidic acid. Although NAPE-PLD is the primary pathway for anandamide synthesis, alternative routes also exist, involving enzymes such as phospholipase C (PLC) and phosphatidylinositol-specific phospholipase C (PI-PLC), which generate intermediate products that can be converted into anandamide. Once synthesized, anandamide levels are tightly regulated by metabolic processes. The primary enzyme responsible for its degradation is FAAH (fatty acid amide hydrolase), which hydrolyzes anandamide into arachidonic acid and ethanolamine, thus deactivating its biological activity. Another enzyme, NAAA (N-acylethanolamine-hydrolyzing acid amidase), also contributes to anandamide degradation, although it mainly targets other lipid amides. In addition to FAAH-mediated hydrolysis, anandamide can undergo oxidative metabolism through enzymes like COX-2 (cyclooxygenase-2), LOX (lipoxygenase) and prostaglandin synthase. These enzymes convert anandamide into various metabolites, some of which may have distinct biological activities [79,80,81,82,83]. Collectively, these enzymatic processes ensure the precise regulation of anandamide levels, balancing its biosynthesis and degradation to maintain homeostasis within the endocannabinoid system.

The term ‘anandamide’ is derived from the Sanskrit word ‘ananda’, which translates as ‘bliss’ or ‘happiness’. This is in reference to its capacity to induce feelings of euphoria. It is a particularly intriguing compound due to its structural and functional similarities to THC (tetrahydrocannabinol), the primary psychoactive constituent of cannabis, yet its endogenous synthesis occurs within the human body [82].

Anandamide has been demonstrated to elicit a number of the biological effects observed in plant-derived cannabinoids, including those affecting the cardiovascular system, which is a characteristic consequence of their recreational use [83]. Anandamide has been demonstrated to elicit a prolonged hypotensive response in anesthetized animals, whereby sympathetic activity at nerve endings in the heart and blood vessels is inhibited. In conscious rats, anandamide has been observed to induce bradycardia and a transient increase in blood pressure, which is followed by long-lasting pressure effects associated with renal and mesenteric vasoconstriction [83,84].

Anandamide has been demonstrated to activate two discrete signaling pathways via the CB1R receptor and the atypical endothelial receptor, designated GPR55. The latter is associated with integrin activation and clustering in human endothelial cells. This process involves the translocation of NFκB via the CB1R receptor, which is mediated by splenic tyrosine kinase, and alternatively, the mobilization of calcium via the PI3K-PLC pathway, which is mediated by the GPR55 receptor. Notably, anandamide has been observed to decrease the production of endothelin-1 and increase nitric oxide levels in human endothelial cells, and this occurs via a mechanism that is independent of the CB1R receptor [85,86].

In their 2007 study, Leweke [87] and colleagues explored how marijuana consumption affects cerebrospinal fluid (CSF) anandamide levels in patients with schizophrenia. They discovered that patients with schizophrenia who used marijuana less frequently had CSF anandamide levels roughly ten times higher than those with higher marijuana use or healthy controls, regardless of plasma anandamide levels. Additionally, there was an inverse relationship between CSF anandamide levels and positive symptoms of schizophrenia. This suggests that frequent marijuana use reduces anandamide signaling in the central nervous system, a pattern not seen in healthy individuals. Furthermore, preliminary evidence points to the endocannabinoid system potentially playing a role in the increased likelihood of substance abuse among schizophrenia patients [88].

Moreover, the molecular mechanisms that regulate the insertion of AEA into the cell membrane of cells with CB1 receptors have been elucidated. These mechanisms, which are linked to the lipid nature of AEA, differ from those associated with classical synaptic receptor activation. AEA displays selectivity towards cholesterol and ceramides, which regulate its biological activity. Cholesterol promotes the transport of AEA to CB1 receptors or intracellular proteins, while ceramides appear after sphingomyelinase activation. AEA can also interact with other membrane proteins. A comprehensive understanding of these mechanisms is essential for the development of endocannabinoid-based treatment strategies [89,90,91,92,93,94,95].

Anandamide (AEA) is a principal endocannabinoid that regulates a multitude of biological processes; however, its action also presents certain drawbacks and limitations. Firstly, the compound is rapidly metabolized by enzymes, resulting in a relatively short-lived effect. Its chemical structure renders it unstable under physiological conditions, which hinders the potential for therapeutic applications. Anandamide exerts its effects on multiple receptors, which may result in undesirable side effects due to a lack of selectivity. Furthermore, its lipophilic properties render it challenging to penetrate cell membranes and the blood–brain barrier, thereby reducing its efficacy. Additionally, it has been associated with adverse effects such as mood changes, impaired motor function and memory impairment. Consequently, the direct medical use of anandamide is currently limited, although research into its modification and action is ongoing to overcome these challenges.

#### 3.2.2. 2-Arachidonoylglycerol (2-AG)

**Figure 12:**
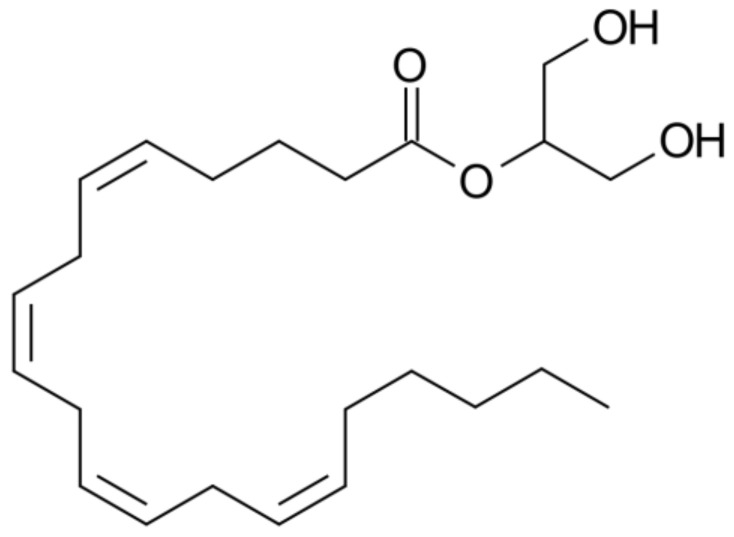
Chemical structure of 2-arachidonoylglycerol.

2-AG, IUPAC name 1,3-Dihydroxypropan-2-yl (5Z,8Z,11Z,14Z)-icosa-5,8,11,14-tetraenoate, is a monoacylglycerol (Figure 12) comprising arachidonic acid and glycerol. It is a lipid compound, which means that it is fat-soluble [96]. 2-AG functions as a ligand for the cannabinoid receptors CB1 and CB2, which are distributed throughout the body, including the brain and the nervous system. The interaction of 2-AG with these receptors modulates the transmission of neural signals, which can affect a range of processes, including pain sensation, appetite regulation and immune responses. The compound was initially identified in the canine gut and characterized as an endogenous ligand for cannabinoid receptors in 1995 [97,98,99].

2-AG binds to CB1 receptors, which are highly expressed in the central nervous system, particularly in the brain. Following its binding to CB1 receptors, 2-AG can modulate the release of neurotransmitters such as GABA (gamma-aminobutyric acid) and glutamate, thereby influencing pain regulation, motor control, memory and other neurological functions [100]. Additionally, 2-AG has been demonstrated to bind to CB2 receptors, which are predominantly expressed in immune cells. Activation of CB2 receptors by 2-AG exerts anti-inflammatory and immunomodulatory effects, which may contribute to the regulation of immune responses and inflammatory processes [101].

GABA (gamma-aminobutyric acid) plays a pivotal role in the modulation of neuronal activity, functioning as the primary inhibitory neurotransmitter in the central nervous system. The action of GABA is to reduce neuronal excitability, thereby decreasing the level of electrical activity in the brain. In the context of the endocannabinoid system, 2-arachidonoylglycerol (2-AG) functions as an endocannabinoid that affects CB1 cannabinoid receptors situated in GABAergic neurons. Activation of these receptors by 2-AG has the potential to result in increased GABA secretion, which may subsequently lead to a reduction in pain sensation and an influence on motor control. An increase in the levels of GABA at GABAergic synapses results in a greater suppression of excitatory neurons, which is crucial for the alleviation of pain and the regulation of motor functions.

Conversely, glutamate is the primary excitatory neurotransmitter in the brain and is responsible for the formation and transmission of synaptic connections, as well as the transfer of information. It is of great importance for the processes of learning, memory and synaptic plasticity. The action of 2-AG on CB1 receptors in glutamatergic neurons has the potential to modulate glutamate release, which in turn affects neuronal activity. The modulation of neuronal activity by 2-AG can result in either a decrease or an increase, depending on the context. This is of particular importance in the regulation of arousal states, cognitive processes and in the mechanisms associated with various neurological and psychiatric disorders.

The interaction of GABA and glutamate in the context of 2-AG action on CB1 receptors exemplifies the intricate complexity of the endocannabinoid system and its profound influence on the equilibrium between excitation and inhibition in the brain. A deeper comprehension of these mechanisms may facilitate the development of novel therapeutic strategies for the management of conditions characterized by neurotransmission imbalances, including depression, anxiety and neuropathic pain [100,101].

2-AG is synthesized within the body primarily through the hydrolysis of membrane phospholipids. This process is regulated by a number of enzymes, of which phospholipases play a pivotal role. Phospholipase C (PLC) is responsible for the degradation of phosphatidylinositol 4,5-bisphosphate (PIP2) to diacylglycerol (DAG), which is subsequently converted to 2-AG by the enzyme diacylglycerol lipase (DAGL) [102,103]. Following the completion of its function, 2-AG is degraded by hydrolytic enzymes, predominantly monoacylglycerol lipase (MAGL). MAGL facilitates the conversion of 2-AG into arachidonic acid and glycerol. Another enzyme that may be involved in 2-AG degradation is fatty acid amide hydrolase (FAAH), although its primary function is the degradation of anandamide (AEA) [104].

In the context of research on the endocannabinoid system, endocannabinoid-degrading enzymes such as FAAH (fatty acid amide hydrolase) have emerged as key therapeutic targets. FAAH is responsible for the hydrolysis of anandamide, a naturally occurring cannabinoid, which affects the levels of endocannabinoids in the brain. Inhibition of FAAH has been demonstrated to increase the availability of anandamide, with beneficial effects in the treatment of a range of conditions, including chronic pain, anxiety and depression. One example of a FAAH inhibitory compound is BIA-10-2474, which was developed as a potential analgesic drug. It is regrettable that BIA-10-2474 did not progress beyond the clinical trial phase due to the emergence of significant adverse effects during the course of the trials. In 2016, during the phase I trial, a number of unforeseen adverse reactions occurred in participants, resulting in the hospitalization of several individuals and, ultimately, the death of one participant. This incident emphasized the necessity for a comprehensive evaluation of the safety and side effects of new compounds in this category. In light of these setbacks, researchers have concentrated their efforts on the creation of new, safer FAAH inhibitors. This process involves the identification of compounds that demonstrate enhanced selectivity towards FAAH and a reduced propensity to induce adverse effects. Over the past few years, numerous novel compounds have been developed that exhibit more favorable safety profiles [104].

The latest advances in this pharmacological category encompass a number of pivotal elements. Firstly, there is a necessity to develop compounds that are more selective for FAAH, thereby minimizing the effects on other enzymes and metabolic pathways. Such an approach may prove an effective means of reducing the risk of adverse effects. Secondly, enhancements to the pharmacokinetic profile are crucial. This encompasses enhanced bioavailability, an extended duration of action and diminished interaction with other pharmaceutical agents, which may result in superior tolerability. Another crucial aspect is the utilization of novel formulation techniques. The utilization of novel carriers and drug delivery strategies, such as nanoparticles or liposomes, has the potential to enhance the stability and efficacy of these compounds. Furthermore, the development of biomarkers that can predict the response to therapy and individualize treatment is a noteworthy area of research. This will facilitate the identification of patients who will derive the greatest benefit from FAAH inhibitory therapy. It is imperative that preclinical studies are intensified. It is imperative that these studies facilitate a more comprehensive understanding of the mechanisms of action of the novel compounds and their potential side effects prior to the commencement of clinical trials.

The available evidence indicates that modulation of the endocannabinoid system, including the increase in 2-AG levels, may prove beneficial in the treatment of chronic pain and inflammatory conditions such as arthritis. The elevation of 2-AG levels may facilitate enhanced pain regulation and a reduction in inflammation, thereby providing relief for patients afflicted with chronic pain and inflammatory conditions [105]. The endocannabinoid system (ECS) plays a pivotal role in neuroprotection, which may prove to be a valuable avenue for the treatment of neurodegenerative diseases such as Alzheimer’s and Parkinson’s disease. An increase in 2-AG levels may facilitate the protection of nerve cells from damage and degeneration. By modulating inflammatory responses and oxidative stress, 2-AG may assist in maintaining the health of nerve cells and slowing the progression of neurodegenerative conditions [106,107,108,109]. 2-AG and other endocannabinoids have been demonstrated to exert an influence on mood and stress responses. Research on the endocannabinoid system (ECS) indicates that modulating its action may facilitate the development of novel therapeutic interventions for anxiety disorders, depression and post-traumatic stress disorder (PTSD). A deeper comprehension of the influence of endocannabinoids on the regulation of emotions and stress responses may facilitate the development of more efficacious treatments for these conditions [110,111]. The modulation of CB1 receptors by endocannabinoids has implications for appetite and metabolism, which may be important in the treatment of obesity and metabolic syndrome. Endocannabinoids, such as 2-AG, interact with CB1 receptors in the brain and other parts of the body to regulate appetite and metabolic processes. An understanding of these mechanisms may facilitate the development of novel therapeutic strategies to control appetite, improve metabolism and, ultimately, support weight management and the treatment of metabolic disorders [112,113].

The cannabinoid receptors CB1 and CB2 have been demonstrated to influence a wide range of physiological functions. Given its role in regulating pain, inflammation, mood, memory and other functions, 2-AG and the endocannabinoid system are the subject of considerable research into their potential therapeutic applications in the treatment of various diseases. The research into the synthesis and degradation of 2-AG has led to a more comprehensive understanding of its role within the body. A deeper comprehension of these mechanisms may facilitate the creation of novel therapeutic modalities with potential applications in the management of conditions such as chronic pain, mood disorders and inflammatory and neurodegenerative diseases.

## 4. Conclusions

Phytocannabinoids and endocannabinoids represent two principal groups of chemical compounds that interact with the endocannabinoid system in mammalian bodies, including humans. The endocannabinoid system (ECS) constitutes a network of receptors, enzymes and ligands that plays a pivotal role in the regulation of a range of physiological processes, including pain sensation, appetite, mood, memory, motility and immune system function.

Phytocannabinoids are naturally occurring compounds that are found in a variety of plants, with hemp (Cannabis sativa) being a particularly notable source. The most well-known phytocannabinoids are tetrahydrocannabinol (THC), which has psychoactive effects, and cannabidiol (CBD), which has no psychoactive effects but has broad therapeutic potential. A number of other phytocannabinoids, including CBN, CBG and CBC, have also been identified and are understood to have significant biological effects.

Endocannabinoids are compounds that are produced endogenously by mammalian organisms. The most well-studied endocannabinoids are anandamide (AEA) and 2-arachidonoylglycerol (2-AG). These compounds act as neuromodulators by binding to the cannabinoid receptors CB1 and CB2, which are distributed throughout the body, but are particularly abundant in the brain and immune system.

Both groups of compounds, phytocannabinoids and endocannabinoids, interact with the ECS by binding to CB1 and CB2 receptors, thereby affecting a variety of bodily functions, including pain relief and modulation of the immune response. This intricate interplay renders the ECS an invaluable subject of investigation with regard to the treatment of a multitude of ailments, including chronic pain, neurodegenerative disorders, inflammation and psychiatric disorders.

The legislative framework governing cannabis research and utilization varies considerably between countries, presenting a significant challenge for researchers and industry stakeholders. A considerable number of countries have implemented restrictive regulations pertaining to the cultivation, testing and utilization of cannabis, which constrains the ability to undertake large-scale clinical trials. Although there is considerable preliminary evidence of the potential health benefits of phytocannabinoids, there is a paucity of large-scale, controlled clinical trials that can unequivocally confirm their efficacy and safety in various medical applications. The endocannabinoid system is intricate and not yet fully elucidated. Further research is required to gain a comprehensive understanding of the impact of phytocannabinoids and endocannabinoids on this system and to ascertain their potential for therapeutic applications. The response to cannabinoids can vary significantly between patients due to a number of factors, including genetic differences, health status, diet, medications taken and other variables. This can present challenges in standardizing dosage and predicting the response to therapy. Furthermore, cannabinoids have been observed to induce adverse effects, including drowsiness, dizziness and cognitive impairment.

Additionally, they can interact with other pharmaceutical agents, potentially impacting their efficacy or leading to adverse effects. In conclusion, while phytocannabinoids and endocannabinoids have significant therapeutic potential, research into them is confronted with numerous challenges. Further research and regulatory changes are essential to facilitate a deeper comprehension of these compounds and their optimal utilization.

In conclusion, it is hoped that this review will enhance the understanding and perspective on the use of cannabinoids.

## Data Availability

Data are contained within the article.
